# The comprehensive role of E-cadherin in maintaining prostatic epithelial integrity during oncogenic transformation and tumor progression

**DOI:** 10.1371/journal.pgen.1008451

**Published:** 2019-10-28

**Authors:** Adam Olson, Vien Le, Joseph Aldahl, Eun-Jeong Yu, Erika Hooker, Yongfeng He, Dong-Hong Lee, Won Kyung Kim, Robert D. Cardiff, Joseph Geradts, Zijie Sun

**Affiliations:** 1 Department of Cancer Biology, Beckman Research Institute, City of Hope, Duarte, California, United States of America; 2 Center for Comparative Medicine, University of California at Davis, Davis, California, United States of America; 3 Department of Population Sciences, Beckman Research Institute, City of Hope, Duarte, California, United States of America; Stanford University School of Medicine, UNITED STATES

## Abstract

E-cadherin complexes with the actin cytoskeleton via cytoplasmic catenins and maintains the functional characteristics and integrity of the epithelia in normal epithelial tissues. Lost expression of E-cadherin disrupts this complex resulting in loss of cell polarity, epithelial denudation and increased epithelial permeability in a variety of tissues. Decreased expression of E-cadherin has also been observed in invasive and metastatic human tumors. In this study, we investigated the effect of E-cadherin loss in prostatic epithelium using newly developed genetically engineered mouse models. Deletion of E-cadherin in prostatic luminal epithelial cells with modified probasin promoter driven *Cre* (PB-Cre4) induced the development of mouse prostatic intraepithelial neoplasia (PIN). An increase in levels of cytoplasmic and nuclear β-catenin appeared in E-cadherin deleted atypical cells within PIN lesions. Using various experimental approaches, we further demonstrated that the knockdown of E-cadherin expression elevated free cytoplasmic and nuclear β-catenin and enhanced androgen-induced transcription and cell growth. Intriguingly, pathological changes representing prostatic epithelial cell denudation and increased apoptosis accompanied the above PIN lesions. The essential role of E-cadherin in maintaining prostatic epithelial integrity and organization was further demonstrated using organoid culture approaches. To directly assess the role of loss of E-cadherin in prostate tumor progression, we generated a new mouse model with bigenic *Cdh1* and *Pten* deletion in prostate epithelium. Early onset, aggressive tumor phenotypes presented in the compound mice. Strikingly, goblet cell metaplasia was observed, intermixed within prostatic tumor lesions of the compound mice. This study provides multiple lines of novel evidence demonstrating a comprehensive role of E-cadherin in maintaining epithelial integrity during the course of prostate oncogenic transformation, tumor initiation and progression.

## Author summary

The biological significance of E-cadherin in maintaining prostatic epithelial integrity and related molecular mechanisms are still unclear. In this study, using mouse genetic tools, we directly address this important and unresolved question. Conditional deletion of E-cadherin in mouse prostatic epithelia resulted in prostatic intraepithelial neoplasia (PIN) development but no prostatic tumor formation. Both *in vivo* and *in vitro* data showed that loss of E-cadherin modulates the cellular localization of β-catenin, elevates its cytoplasmic and nuclear levels, and enhances its activity in transcription and cell proliferation. Intriguingly, in addition to PIN lesions, increased epithelial denudation and cell apoptosis also appeared within PIN lesions. This implicates that although lost E-cadherin is sufficient to introduce oncogenic transformation in prostatic epithelia, it also induces cell apoptosis and disrupts epithelial structure, preventing atypical PIN cells from progressing to tumor cells. Simultaneous deletion of *Pten*, a tumor suppressor, and E-cadherin in prostatic epithelia resulted in early onset, invasive prostatic tumors with admixture of goblet cells. These results demonstrate a critical role of E-cadherin in promoting prostatic tumor transdifferentiation and progression. This study further elucidates the dynamic role of E-cadherin in maintaining prostatic epithelial integrity during tumor initiation and progression.

## Introduction

Maintaining the integrity of cell-cell contacts is essential in the development of multicellular organisms. Cadherins are key regulators in this biological event and play a critical role in morphogenic processes during development [1–3]. E-cadherin, coded by *Cdh1*, is the founder member of the cadherin superfamily and complexes with actin cytoskeleton and cytoplasmic catenin molecules in order to maintain the functional characteristics and integrity of epithelia [4,5]. Disruption of the E-cadherin mediated complex, due primarily to lost or decreased expression of E-cadherin, resulted in epithelial abnormalities and severe developmental defects in a variety of tissues and organs [6–8]. Conditional deletion of the *Cdh1* gene in mouse mammary glands disrupts terminal differentiation and results in massive cell death in mutant mammary glands [9]. Similarly, temporal deletion of E-cadherin in Nkx3.1 expressing cells in prostatic epithelium induces apoptotic cell death via anoikis, which subsequently promotes vertical divisions from prostatic basal to luminal cells and increases luminal cell growth and expansion [10].

Aberrant expression and mutations in the *CDH1* gene have been observed in many human epithelial tumors [11]. Loss or reduction of E-cadherin expression appears in many advanced, poorly differentiated, and invasive human tumors, suggesting that reducing cell-cell contacts mediated by E-cadherin promotes tumor progression and metastasis [12,13]. It has been shown that aberrant E-cadherin expression in tumor cells dysregulates the cytoplasmic pools of β-catenin and enhance its activity in transcription [14]. Cellular levels of β-catenin are tightly regulated in normal cells and aberrant increased β-catenin expression has been closely corroborated in oncogenic transformation during the course of tumor initiation [15]. Mutations in both β-catenin and its destruction complex components can increase nuclear β-catenin levels, have been observed in many tumors and are directly associated with human tumorigenesis [15,16]. However, mutations in β-catenin, APC, and other components of the destruction complex appear very rarely in prostate cancer cells [17–19], suggesting that other regulatory mechanisms underlie the activation of Wnt/β-catenin signaling in promoting prostate tumorigenesis.

In this study, we assessed the critical role of E-cadherin in prostate development and tumorigenesis using mouse genetic tools. Conditional deletion of E-cadherin in mouse prostatic epithelial cells induces the development of mouse prostatic intraepithelial neoplasia (PIN). An increase in cytoplasmic and nuclear β-catenin, and its activity in transcription and cell proliferation were observed in E-cadherin deleted cells in both *in vivo* and *in vitro* experiments. However, no prostatic tumors were observed in the E-cadherin mutant mice. Intriguingly, in addition to oncogenic transformation and PIN formation, loss of cell-cell adhesion and prostatic epithelial structure as well as elevated epithelial denudation and cell apoptosis appeared in prostate tissues of the mutant mice. Observations of these distinct cellular events in prostatic epithelia suggest that although lost E-cadherin is sufficient to introduce oncogenic transformation, it also induces cell apoptosis and disrupts epithelial structure and cell-cell contacts which in turn prevents atypical PIN cells from progressing to tumor cells. Using the newly generated Pten and E-cadherin compound knockout mice, we further investigated the effect of E-cadherin loss in prostate tumor progression. Early onset and invasive prostatic tumors with an admixture of goblet cells were observed in the prostate of compound mice. Our findings provide fresh insight into the comprehensive role of E-cadherin in maintaining epithelial integrity during prostate tumor initiation and progression.

## Results

### Conditional deletion of E-cadherin expression in prostatic luminal cells

**Fig 1:**
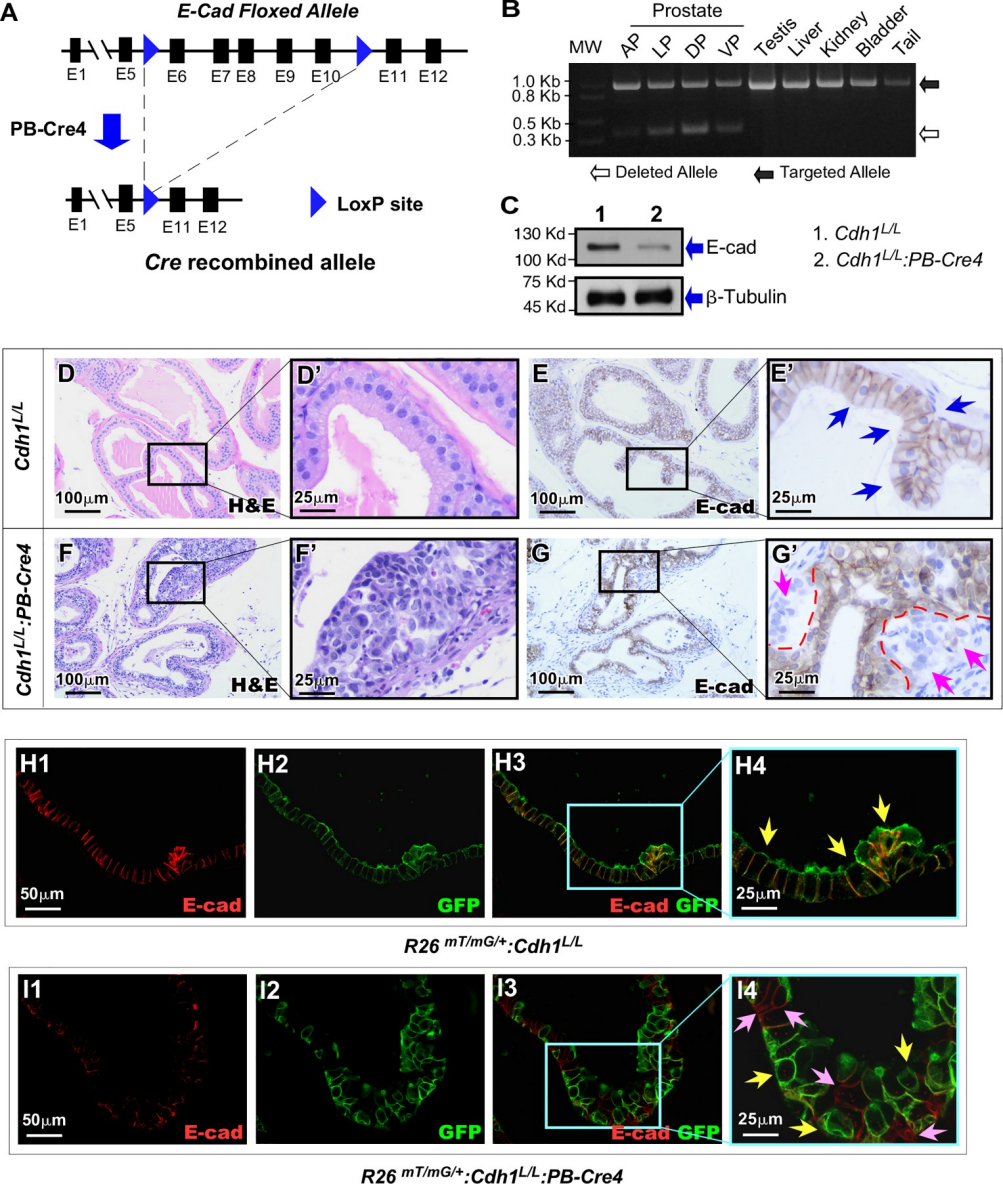
Generation of Probasin driven prostate specific deletion of E-cadherin in mice. **A.** A scheme was shown of the targeted *Cdh1* allele undergoing Probasin driven Cre mediated recombination. **B.** Recombination PCR with DNA isolated from *Cdh1*^*L/L*^:*PB-Cre4* anterior prostate (AP), lateral prostate (LP), dorsal prostate (DP), ventral prostate (VP) and other tissues as indicated. Band sizes correspond to the DNA ladder in the left lane. **C.** Western blot results from protein lysates isolated from prostate tissues of *Cdh1*^*L/L*^: *PB-Cre4* and *Cdh1*^*L/L*^ control mice. **D-G’.** Representative H&E & E-cadherin IHC staining of prostate tissue sections from *Cdh1*^*L/L*^:*PB-Cre4* and *Cdh1*^*L/L*^ mice. Blue arrows indicate E-cadherin positive epithelium (E’). Pink arrows indicate E-cadherin negative regions with dotted lines marking the border between E-cadherin negative cells and surrounding E-cadherin positive epithelium (G’). **H1-I4.** Representative images of Co-IF staining of mouse prostate sections displaying overlay of E-cadherin and mGFP (yellow arrows, H4) in *R26*^*mTmG/+*^*; PB-Cre4* control mice. Lost/reduced E-cadherin staining is observed in mGFP+ (yellow arrows, I4) cells of *R26*^*mTmG/+*^:*Cdh1*^*L/L*^:*PB-Cre4* mice. Cells without mGFP expression retain E-cad positivity in *R26*^*mTmG/+*^:*Cdh1*^*L/L*^:*PB-Cre4* mice (pink arrows, I4). Scale bars, located in the bottom left corner of images, are sized as indicated.

E-cadherin plays an essential role in maintaining epithelial integrity, and loss or reduction of E-cadherin expression has been linked to abnormal development and tumorigenesis [3,9,12,20]. To directly assess the effect of E-cadherin loss in prostate epithelia, we generated a conditional *Cdh1* deletion mouse line, *Cdh1*^*L/L*^:*PB-Cre4*, in which conditional deletion of the *Cdh1* allele is regulated through *Cre* expression by a modified probasin promoter, ARR2PB, in prostatic luminal epithelia (Fig 1A). To confirm *PB-Cre4* mediated recombination, genomic DNA samples were isolated from the four different prostate lobes (anterior, dorsal, lateral and ventral) individually as well as from the testes, liver, kidney, bladder and tail of a *Cdh1*^*L/L*^:*PB-Cre4* mouse (Fig 1B). Although an approximately 980 bp PCR fragment representing floxed *Cdh1* alleles appeared in all of the above mouse tissues, an approximately 350 bp PCR fragment, corresponding to the deletion of exons 6 through 10 of *Cdh1* alleles, was only observed in prostatic lobes (Fig 1B), suggesting specific deletion of E-cadherin in prostate tissues. Reduction of E-cadherin protein expression was observed in prostate tissue samples of *Cdh1*^*L/L*^:*PB-Cre4* mice by western blotting (Lane 2 in Fig 1C). The reduction of E-cadherin expression was further assessed by immunohistochemistry (IHC) in both *Cdh1*^*L/L*^:*PB-Cre4* and control mice. Typical prostatic glandular structure appeared in tissue samples isolated from *Cdh1*^*L/L*^ mice (Fig 1D and 1D’) with uniform cellular membrane staining of E-cadherin in luminal epithelial cells (blue arrows, Fig 1E and 1E’). However, pathologic changes similar to prostatic hyperplasia and prostatic intraepithelial neoplasia, PIN, presented in prostate tissues of *Cdh1*^*L/L*^:*PB-Cre4* mice (Fig 1F and 1F’). Intriguingly, loss of E-cadherin expression appeared in a portion of prostatic epithelia, mainly within PIN lesions. These E-cadherin negative cells appeared to cluster between the basement membrane and a layer of epithelium with intact E-cadherin, reminiscent of pagetoid spread (pink arrows, Fig 1G’). The above observation of partial E-cadherin deletion in prostate epithelia is new and intriguing. The pagetoid distribution of E-cadherin negative cells is very similar histologically to reports of E-cadherin negative lobular carcinoma *in situ* (LCIS) of the breast [21,22]. To confirm that loss of E-cadherin expression is indeed a direct result of *Cre* mediated recombination, we generated a new mouse strain, *R26*^*mTmG/+*^:*Cdh1*^*L/L*^:*PB-Cre4*, in which the change in expression from membrane-targeted tandem dimer tomato (mT) to membrane-targeted green fluorescence protein (mGPF) and deletion of *Cdh1* occur simultaneously through *Cre* mediated recombination (S1 Fig.). Co-immunofluorescence staining (Co-IF) showed clear overlay of E-cadherin and mGFP proteins in prostate epithelial cells of *R26*^*mTmG/+*^:*PB-Cre4* control mice (yellow arrows, Fig 1H3-4). In contrast, epithelial cells with either GFP or E-cadherin staining presented in prostate samples of *R26*^*mTmG/+*^:*Cdh1*^*L/L*^:*PB-Cre4* mice (yellow or pink arrows respectively, Fig 1I3-4), indicating a mosaic recombination occurring in mouse prostatic epithelia. These data not only demonstrate the deletion of E-cadherin in prostatic epithelium through Probasin driven *Cre* expression but also elucidate the mosaic nature of this recombination event in the prostate of *Cdh1*^*L/L*^:*PB-Cre4* mice.

### Loss of E-cadherin expression in prostatic luminal cells induces prostatic intraepithelial neoplasia formation

**Fig 2:**
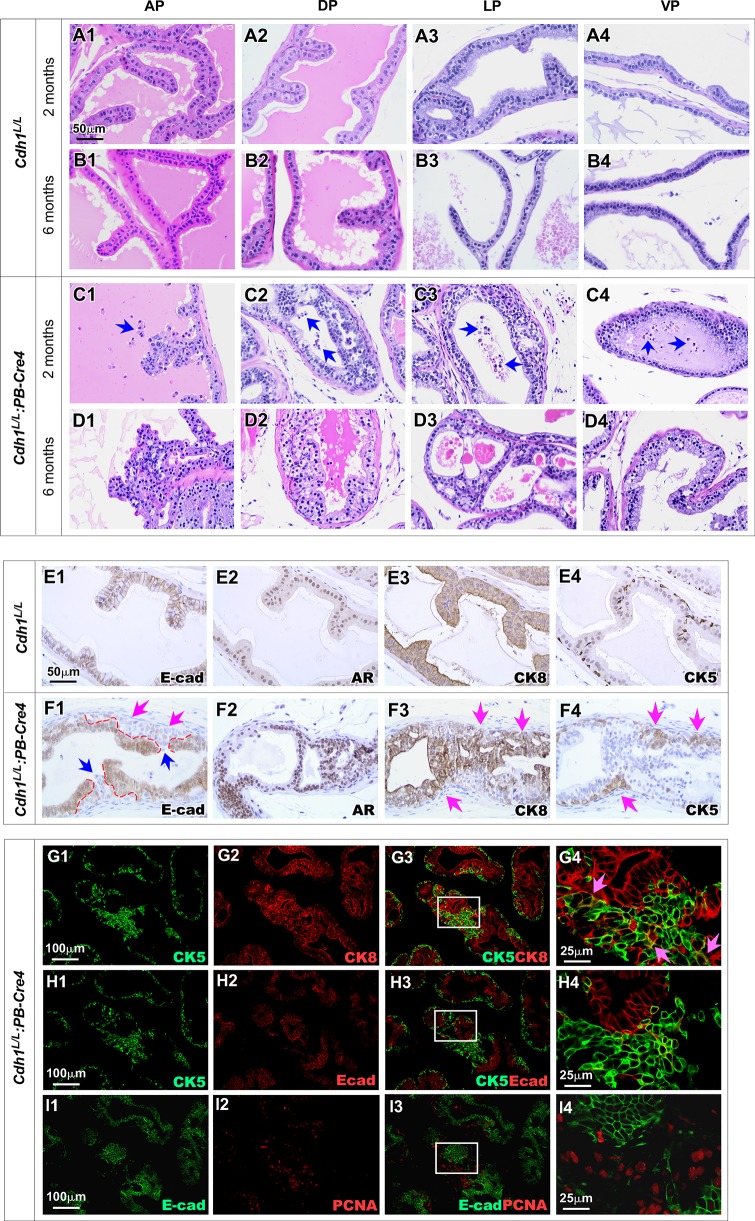
Prostate specific E-cadherin knockout results in PIN with Pagetoid Features. **A1-D4.** Representative H&E images of prostate sections from mice of the indicated genotypes and ages. Blue arrows indicate cells observed sloughing off into the lumen (C1-C4). **E1-F4.** Representative images of IHC staining for the indicated proteins in prostate tissue sections of *Cdh1*^*L/L*^:*PB-Cre4* & *Cdh1*^*L/L*^ mice. Pink arrows indicate clusters of E-cadherin null cells (F1). The interface between E-cadherin positive and E-cadherin negative cells is shown with red dashed lines (F1). PIN lesions are indicated with pink arrows F3 and F4. **G1-I4.** Representative images of CoIF staining for various antibodies as indicated. CK5 and CK8 positive intermediate cells are indicated with pink arrows (G4). Scale bars, located in the bottom left corner of images, are sized as indicated.

The *Cdh1*^*L/L*^:*PB-Cre4* offspring were born at the expected Mendelian ratios and appeared normal with no obvious differences from their *Cdh1*^*L/L*^ controls and wild-type littermates at birth. Per the recommendations of the Mouse Models of Human Cancers Consortium Prostate Pathology Committee [23], we assessed *Cdh1*^*L/L*^:*PB-Cre4* mice from birth and identified pathological lesions resembling low grade PIN starting at about 6 weeks. Prostate tissues isolated from two-month-old *Cdh1*^*L/L*^:*PB-Cre4* mice showed clear PIN lesions in both the DP and LP regions (Fig 2C1-4). In addition to PIN lesions, an increase in detached epithelial cells also presented in lumen of prostatic glands in the above tissues (blue arrows Fig 2C1-4). At 6 months of age, *Cdh1*^*L/L*^:*PB-Cre4* mice showed similar PIN lesions present in all four prostate lobes but with fewer detached cells (Fig 2D1-4). No abnormalities were observed in sex and age matched *Cdh1*^*L/L*^ control mice (Fig 2A1-4 and 2B1-4). These observations demonstrate that the deletion of E-cadherin induces prostatic epithelial cell oncogenic transformation and PIN formation. However, continuing examining old aged *Cdh1*^*L/L*^:*PB-Cre4* mice, showed no or slow progression of PIN lesions with age and no prostate tumors were observed in mice up to 12 months of age (Table 1). These data demonstrate conditional deletion of E-cadherin in prostatic luminal epithelia is insufficient to promote PIN to progress to prostate cancer, suggesting that other cellular events may co-occur and play a tumor suppressive role during the course of E-cadherin loss in prostatic epithelia.

**Table 1:** Pathological abnormalities in the prostates of *Cdh1*^*L/L*^:*PB-Cre4* mice.

Prostate Lobe	< 2 Months	2–4 Months	4–12 Months
Anterior Prostate	2 of 4 Normal 2 of 4 LGPIN*	1 of 5 Normal 3 of 5 Hyperplasia 1 of 5 LGPIN	1 of 5 Normal 2 of 5 Hyperplasia 2 of 5 LGPIN
Dorsal Prostate	1 of 4 Normal 3 of 4 LGPIN	2 of 5 Hyperplasia 3 of 5 LGPIN	1 of 5 Hyperplasia 4 of 5 LGPIN
Lateral Prostate	1 of 4 Normal 1 of 4 Hyperplasia 2 of 4 LGPIN	2 of 5 Hyperplasia 3 of 5 LGPIN	1 of 5 Hyperplasia 4 of 5 LGPIN
Ventral Prostate	1 of 4 Normal 3 of 4 Hyperplasia	1 of 5 Normal 2 of 5 Hyperplasia 2 of 5 LGPIN	3 of 5 Normal 1 of 5 Hyperplasia 1 of 5 LGPIN

*LGPIN: Low grade prostatic intraepithelial neoplasia

Using immunohistochemical (IHC) approaches, we further evaluated the cellular properties of atypical cells within the above PIN lesions. A cluster of E-cadherin negative cells was present under a layer of E-cad expressing epithelial cells, consistent with pagetoid distribution as described earlier (pink arrows, Fig 2F1, also see Fig 1G’). Most atypical cells also showed positive staining for AR, and CK8, a prostatic luminal cell marker (Fig 2F2 and 2F3). Intriguingly, an increase in CK5 positive cells was observed within the above PIN lesions. CK5 and CK8 positive staining was observed in the same areas in adjacent tissue sections (pink arrows, Fig 2F3 and 2F4), suggesting that those cells express both CK5 and CK8, possessing intermediate cellular properties. In this study, we also observed the anoikis of E-cadherin negative cells that were spilled into the lumen from regions where, at the epithelial apical surface, the layer of E-cadherin positive cells was disrupted (blue arrows, Fig 2F1). Using Co-IF approaches, we examined if prostatic epithelial cell anoikis can induce luminal cell replenishment through increased differentiation from basal cells and subsequent cell proliferation [10]. A significant increase in intermediate epithelial cells with both CK5 and CK8 positive staining (pink arrows, Fig 2G4) was observed within PIN lesions in comparison with the adjacent epithelia. The majority of these intermediate cells appeared negative for E-cadherin staining (Fig 2H1-4) but showed high levels of positive staining for PCNA (Fig 2I1-4), suggesting their newly generated and proliferative nature. These observations demonstrate that luminal epithelial denudation and subsequent basal to luminal epithelial replenishment co-occur during the course of PIN lesion development in E-cadherin deletion epithelia. These data also implicate that while deletion of E-cadherin increases proliferative properties of atypical cells, it also impairs their ability to maintain cell-cell contacts resulting in detachment from the epithelium and restricting progression to prostatic tumor lesions.

### Deletion of E-cadherin increases the cytoplasmic and nuclear localization as well as the transcriptional activity of β-catenin in prostate cancer cells

**Fig 3:**
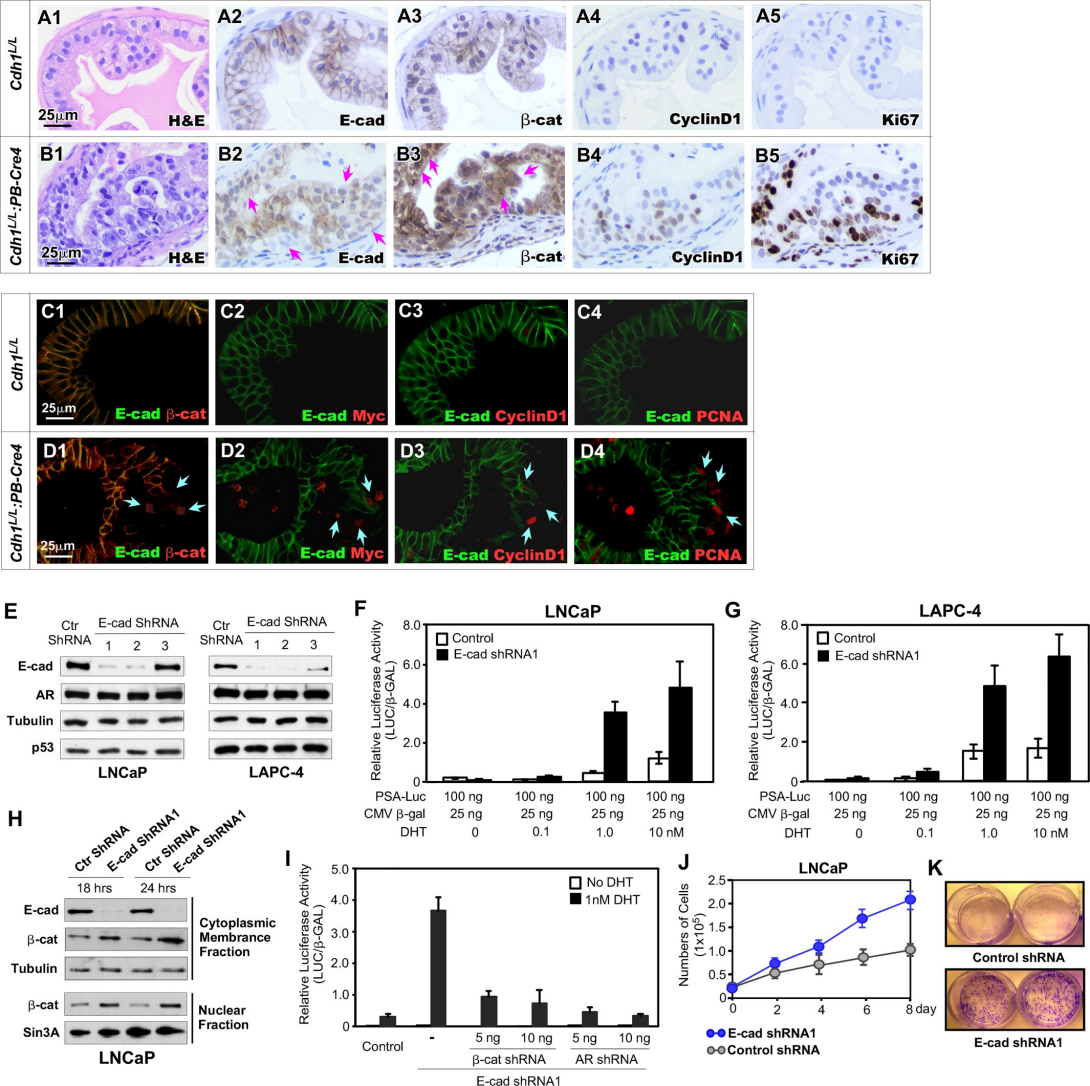
Loss of E-cadherin induces Wnt/β-catenin signaling activation in prostate cancer cells. **A1-B5**. Representative H&E and IHC images of adjacent prostate tissue sections from *Cdh1*^*L/L*^ and *Cdh1*^*L/L*^:*PB-Cre4* mice stained with antibodies as indicated. E-cadherin negative PIN cells (pink arrows, B2) display increased cytoplasmic and nuclear staining for β-cat (pink arrows, B3). **C1-D4**. Representative images of Co-IF staining of adjacent prostate tissue sections from *Cdh1*^*L/L*^:*PB-Cre4* and *Cdh1*^*L/L*^ mice. Cells with detectable expression for Wnt-signaling proteins (β-cat, Myc, CyclinD1) and proliferation marker PCNA are indicated with green arrows (D1-D4). **E.** Western blot results for LNCap & LAPC4 cell lines treated with either control or one of three E-cadherin ShRNA lentiviruses. **F-G.** Relative PSA-luciferase activity observed in LNCaP and LAPC4 cells treated with control and E-cadherin ShRNA1 with increasing concentrations of DHT in the medium. **H.** Western blot results from LNCaP cells separated into cytoplasmic/membrane & nuclear fractions following treatment with control or E-cadherin ShRNA1 for 18 and 24 hrs. **I.** Relative luciferase activity in LNCaP cells treated with E-cadherin ShRNA1 in addition to indicated concentrations of β-cat and AR ShRNAs. All conditions were performed both in the presence and absence of DHT. **J.** MTS assay results representing the number of LNCaP cells for 8 days with control or E-cad ShRNA1 treatments as indicated. **K.** Images of two wells from each group as indicated after a 12-day colony formation assay. Images were taken following fixation and staining with crystal violet. Scale bars, located in the bottom left corner of images, are sized as indicated.

Previous studies have shown that mutations in β-catenin, APC, and other components of the destruction complex appear very rarely in prostate cancer cells [17–19], implicating other regulatory mechanisms for activating Wnt/β-catenin signaling in promoting prostate tumorigenesis. Disruption of E-cadherin-involved protein complexes with the actin cytoskeleton and catenins has been frequently observed in many human tumor samples including prostate cancers [5,24,25,26]. A regulatory role of E-cadherin in cytoplasmic and nuclear β-catenin has been implicated in prostate cancer cells [27,28]. In this study, we assessed the effect of E-cadherin loss on β-catenin expression and cellular localization using IHC approaches with adjacent tissue sections prepared from *Cdh1*^*L/L*^:*PB-Cre4* and *Cdh1*^*L/L*^ mice. Increased cytoplasmic and nuclear staining of β-catenin was observed in E-cadherin negative atypical cells within PIN lesions of *Cdh1*^*L/L*^:*PB-Cre4* mice (Pink arrows, Fig 3B2-3). Those cells also showed increased staining for Cyclin D1, a transcriptional downstream target of β-catenin, and Ki67 (Fig 3B4-5). In contrast, typical cellular membrane staining for E-cadherin and β-catenin and no or very weak staining for CyclinD1 and Ki67 presented in prostatic epithelial cells in tissue samples of *Cdh1*^*L/L*^ controls (Fig 3A2-5). Using Co-IF approaches, we further examined the loss of E-cadherin in correspondence to the expression of cytoplasmic and nuclear β-catenin within PIN lesions of *Cdh1*^*L/L*^:*PB-Cre4* mice. As shown in Fig 3D1, increased cytoplasmic and nuclear staining of β-catenin appeared in a cluster of E-cadherin null epithelial cells whereas the adjacent E-cadherin positive cells retained typical membrane staining for β-catenin. In adjacent tissues sections, positive staining for Myc, Cyclin D1, and PCNA also appeared in E-cadherin negative cells (blue arrows, Fig 3D2-4). In prostate tissues of *Cdh1*^*L/L*^ control mice, there was overlaid staining on the cellular membrane of both E-cadherin and β-catenin in prostatic epithelial cells and negative staining for Myc, Cyclin D1, and PCNA (Fig 3C1-4). These data demonstrate that loss of E-cadherin expression increases the level of cytoplasmic and nuclear β-catenin which further enhances the transcriptional activity of β-catenin in inducing Cyclin D1 and Myc expression in the prostate of *Cdh1*^*L/L*^:*PB-Cre4* mice.

Observation of increasing expression in β-catenin downstream target genes, Myc and Cyclin D1 in atypical cells bearing E-cadherin loss within PIN lesions in *Cdh1*^*L/L*^:*PB-Cre4* mice suggests a promotional role of β-catenin in oncogenic transformation. It has also been shown that β-catenin is a co-activator of the AR and enhances AR mediated transcription [29,30]. To test if loss of E-cadherin can modulate AR mediated transcription via altered β-catenin levels in prostate cancer cells, we specifically knocked down endogenous E-cadherin expression using small hair-pin RNA interference approaches in human prostate cancer cell lines. As shown in Fig 3E, the knockdown of E-cadherin expression was observed with two shRNA lentiviruses for E-cadherin on both LNCaP and LAPC4 cell lines. To examine the effect of E-cadherin knockdown on AR mediated transcriptional activity, we transfected luciferase reporter plasmids driven by the human prostate specific antigen, PSA, promoter into the above cells. A significant increase in androgen-dependent induction of AR-mediated transcription was observed in samples infected with E-cadherin shRNA lentiviruses (black bars vs. white bars, Fig 3F and 3G). Increased levels of both cytoplasmic and nuclear β-catenin appeared in cells infected with E-cadherin shRNA lentiviruses in comparison to ones infected with control viruses (Fig 3H). Despite increased AR-mediated transcription, AR protein levels remained consistent between mice with and without knockdown of E-cadherin demonstrating that the increase in transcription is the result of β-catenin induced co-activation (Fig 3H). In support of these observations, reducing either β-catenin or AR expression using specific shRNA lentiviruses significantly attenuated androgen-induced AR-mediated transcriptional activity onset by E-cadherin knockdown in cells, providing evidence of a direct role of β-catenin in enhancing AR transcriptional activity in E-cadherin null prostate cancer cells (Fig 3I). To investigate the role of E-cadherin loss in prostate cancer cell growth, LNCaP cells infected with either E-cadherin shRNA or control lentiviruses were examined by the MTS assay. The numbers of cells infected with E-cadherin shRNA lentiviruses were significantly higher after 6- and 8-day culture in comparison to controls (Fig 3J). The growth promoting effect of E-cadherin knockdown in prostate cancer cells was further examined using colony formation assay. Approximately 500 LNCaP cells that were infected with either E-cadherin shRNA or control lentiviruses were seeded, cultured for 12 days, and then fixed and stained with crystal violet. There were more and larger colonies in E-cadherin shRNA infected LNCaP cells than controls (Fig 3K). These data further demonstrate a direct link between deleting E-cadherin expression and elevating cytoplasmic and nuclear β-catenin levels in prostate cancer cells. Given the role of β-catenin as an AR co-activator [31,32], our data further showed that elevated β-catenin promotes AR-mediated transcription and cell growth in E-cadherin deleted prostate cancer cells, elucidating the molecular mechanisms for loss or reduction of E-cadherin in inducing oncogenic transformation and PIN development.

### Deletion of E-cadherin in prostatic epithelium impairs prostatic morphogenesis and prostatic gland formation in organoid culture

**Fig 4:**
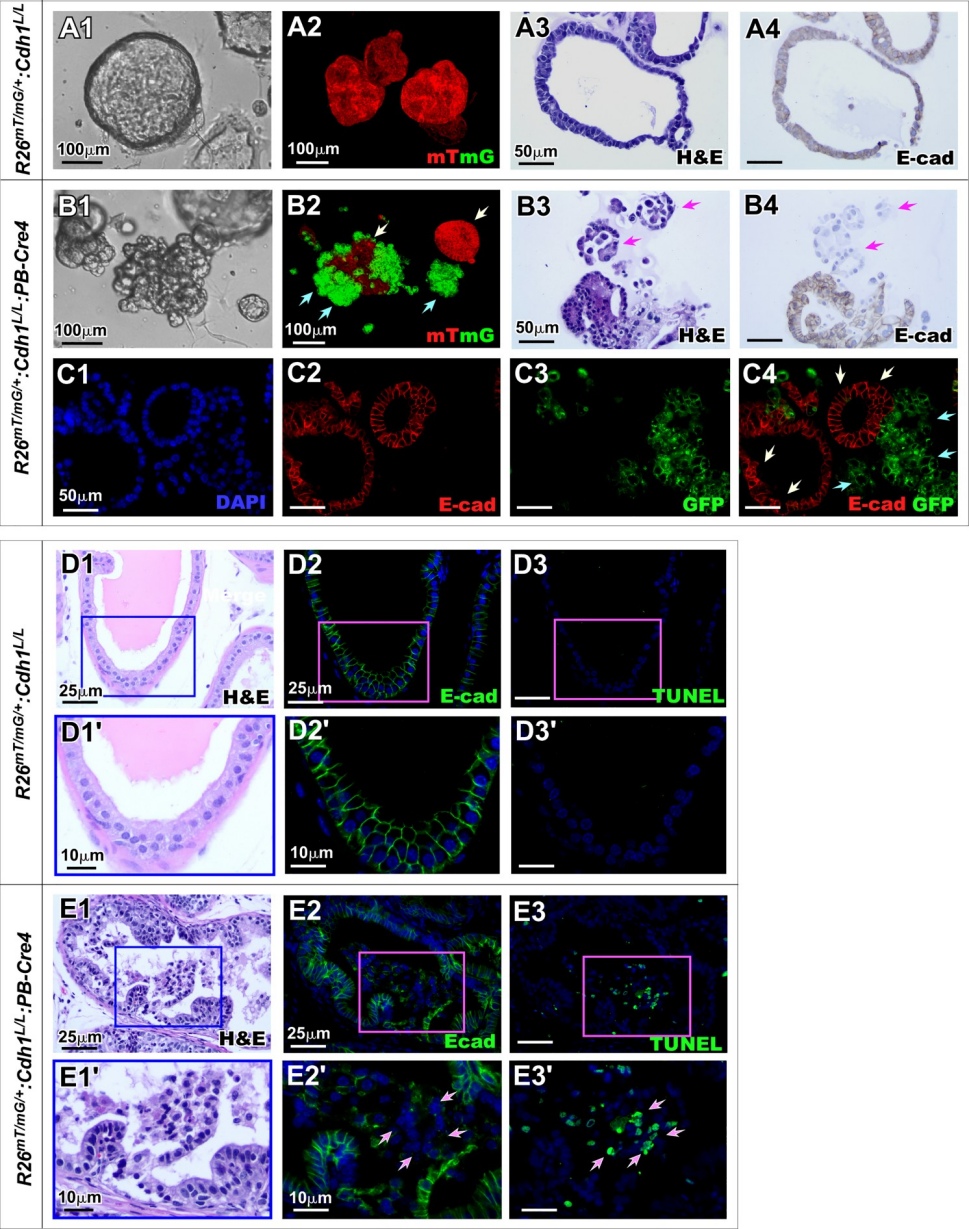
Loss of E-cadherin impedes prostatic epithelial integrity in organoid cultures. **A1-B4.** Representative images comparing organoids established from primary prostate cells from *R26*^*mTmG/+*^:*Cdh1*^*L/L*^:*PB-Cre4* mice and *R26*^*mTmG/+*^:*Cdh1*^*L/L*^ littermate controls. Bright-field images depict organoid morphology observed in each genotype (A1, B1). 3D z-stack images of organoids expressing mTmG reporter fluorescence controlled by Cre recombination (A2 and B2). Green arrows indicate mG positive cells and white arrows indicate mT positive cells due to mosaic recombination events in *R26*^*mTmG/+*^:*Cdh1*^*L/L*^:*PB-Cre4* mice (B2). Representative images of H&E staining and E-cadherin IHC staining in control and E-cadherin KO organoids (A3-A4, B3-B4). Arrows indicate unstructured E-cadherin negative cells separating from organoids (B3-B4). **C1-C4.** Representative fluorescence images of CoIF staining for E-cadherin and GFP in organoids. White arrows indicate mGFP- E-cadherin+ cells while green arrows indicate mGFP+ E-cadherin- cells (C4). **D1-E3’** Representative H&E and fluorescence images of prostate tissue sections from *R26*^*mTmG/+*^:*Cdh1*^*L/L*^:*PB-Cre4* and *R26*^*mTmG/+*^:*Cdh1*^*L/L*^ mice. White arrows indicate E-cadherin negative cells and TUNEL positive cells (E2’ and E3’ respectively). Nuclei were stained with DAPI. Scale bars, located in the bottom left corner of images, are sized as indicated.

To further assess the effect of E-cadherin deletion in the fate of prostatic epithelial cells, we generated organoid cultures using prostatic cells isolated from *R26*^*mTmG/+*^:*Cdh1*^*L/L*^:*PB-Cre4* and *R26*^*mTmG/+*^:*Cdh1*^*L/L*^ mice. After 5 days of culture, organoids derived from prostatic epithelial cells of *R26*^*mTmG/+*^:*Cdh1*^*L/L*^ mice showed normal glandular structure (Fig 4A1), which were fully composed of mT positive cells (Fig 4A2). Histologically typical prostate glands composed of E-cadherin positive epithelial cells presented (Fig 4A3 and 4A4). In contrast, the organoids derived from the prostatic epithelial cells of *R26^mTmG/+^:Cdh1^L/L^:PB-Cre4* mice showed abnormal, unorganized structure (Fig 4B1). Both mT and mGFP expressing cells were observed in the above organoids, suggesting the occurrence of a mosaic recombination (Fig 4B2). Discohesive cellular structures and lost cell-cell contact appeared only in mGFP expressing cells of organoids (green arrows, Fig 4B2). In contrast, organoids comprised of mT positive cells maintained normal smooth-edged organoid structure (white arrows, Fig 4B2). IHC analysis further showed that E-cadherin negative cells formed unstructured cell clusters (arrows, Fig 4B3-4) in contrast with the adjacent E-cadherin positive glandular structures. Using co-IF approaches, we demonstrated that clustered E-cadherin negative and mGFP positive cells were loosely connected and failed to form normal prostate glands (green arrows, Fig 4C4). However, E-cadherin positive but mGFP negative cells formed typical glandular structures in the organoids (white arrows Fig 4C4). These data suggest an irreplaceable role of E-cadherin in prostatic epithelial development, morphogenesis and cell survival despite the concurrent oncogenic capacity introduced by loss of E-cadherin.

To extend our results from prostate organoid cultures, we assessed the effect of E-cadherin deletion in prostatic epithelium using prostate tissue of *Cdh1*^*L/L*^:*PB-Cre4* mice. Clusters of atypical cells appeared loose and unstructured in the lumen of abnormal prostatic glands possessing PIN changes (Fig 4E1). Immunostaining showed that these cells were negatively stained for E-cadherin and positive with TUNEL (Fig 4E2-3’), demonstrating increased apoptosis in these E-cadherin deletion cells. In contrast, prostatic glands from *Cdh1*^*L/L*^ control mice maintained normal, single layered epithelia possessing uniform E-cadherin positive cells with very rare TUNEL staining (Fig 4D1-3’). These results are consistent with our previous observations in prostatic organoid culture and implicate a comprehensive role of E-cadherin deletion in inducing cell apoptosis, which co-occurs with oncogenic transformation simultaneously in the prostate of *Cdh1*^*L/L*^:*PB-Cre4* mice.

### Increased injury repair signaling pathways in prostate epithelium with E-cadherin deletion

**Fig 5:**
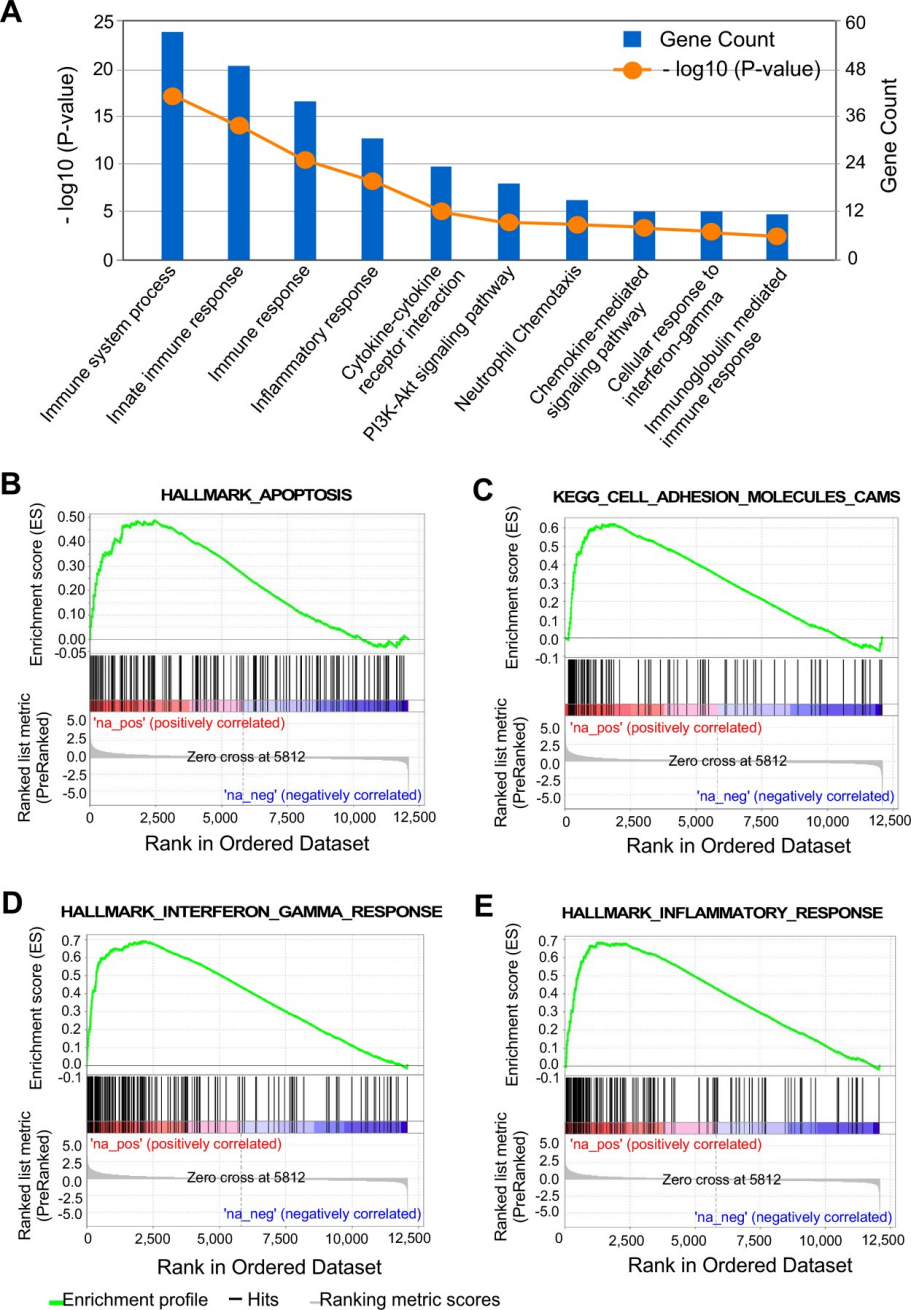
RNA sequencing results indicate increased inflammatory/immune signaling along with increased PI3K-AKT signaling & apoptosis in E-cadherin KO prostate epithelial cells. **A.** DAVID v6.8 results identifying biological processes as being up-regulated in epithelial cells isolated from *Cdh1*^*L/L*^:*PB-Cre4* mice vs. *Cdh1*^*L/L*^ control mice. Gene count indicates the number of genes from each gene set that were upregulated in E-cadherin KO mice compared to controls.–log10 P-value indicates the statistical significance of up-regulation for each pathway. **B-E.** Gene Set Enrichment Analysis (GSEA) results depicting Hallmark and KEGG pathways significantly up-regulated in *Cdh1*^*L/L*^:*PB-Cre4* mice versus *Cdh1*^*L/L*^ control mice. Genes were pre-ranked by p-value for this analysis. All pathways displayed had p-value <0.05.

In search of the molecular basis for the biological role of E-cadherin deletion in the mouse prostate, we analyzed RNA-sequencing data to examine the global transcriptome profiles of *Cdh1*^*L/L*^:*PB-Cre4* versus *Cdh1*^*L/L*^ control mice. Based on 450 upregulated and 182 downregulated DEGs as previously reported (GEO accession: GSE115204) [33], we used DAVID software analysis of Gene Ontology (GO) biological processes and KEGG pathways to identify pathways up and down regulated in prostatic epithelial cells from *Cdh1*^*L/L*^:*PB-Cre4* mice versus littermate controls. Interestingly, pathways related to immune response and inflammation appeared significantly enriched in prostate tissues of *Cdh1*^*L/L*^:*PB-Cre4* mice (Fig 5A). In addition, signaling pathways related to oncogenic transformation and tumorigenesis were also enriched in the above analysis, such as the PI3k-Akt signaling pathway. GSEA analyses of these DEGs showed a similar enrichment of cell signaling pathways related to apoptosis, inflammatory responses, and cell adhesion molecules (Fig 5B–5E). These data suggest that the co-existing oncogenic transformation and cell apoptosis in PIN lesions with E-cadherin negative epithelia induce similar cellular responses as injury including increased apoptosis, inflammation and immune responses.

### Simultaneous deletion of E-cadherin and Pten in prostate epithelium induces earlier onset and more aggressive prostatic carcinomas

**Fig 6:**
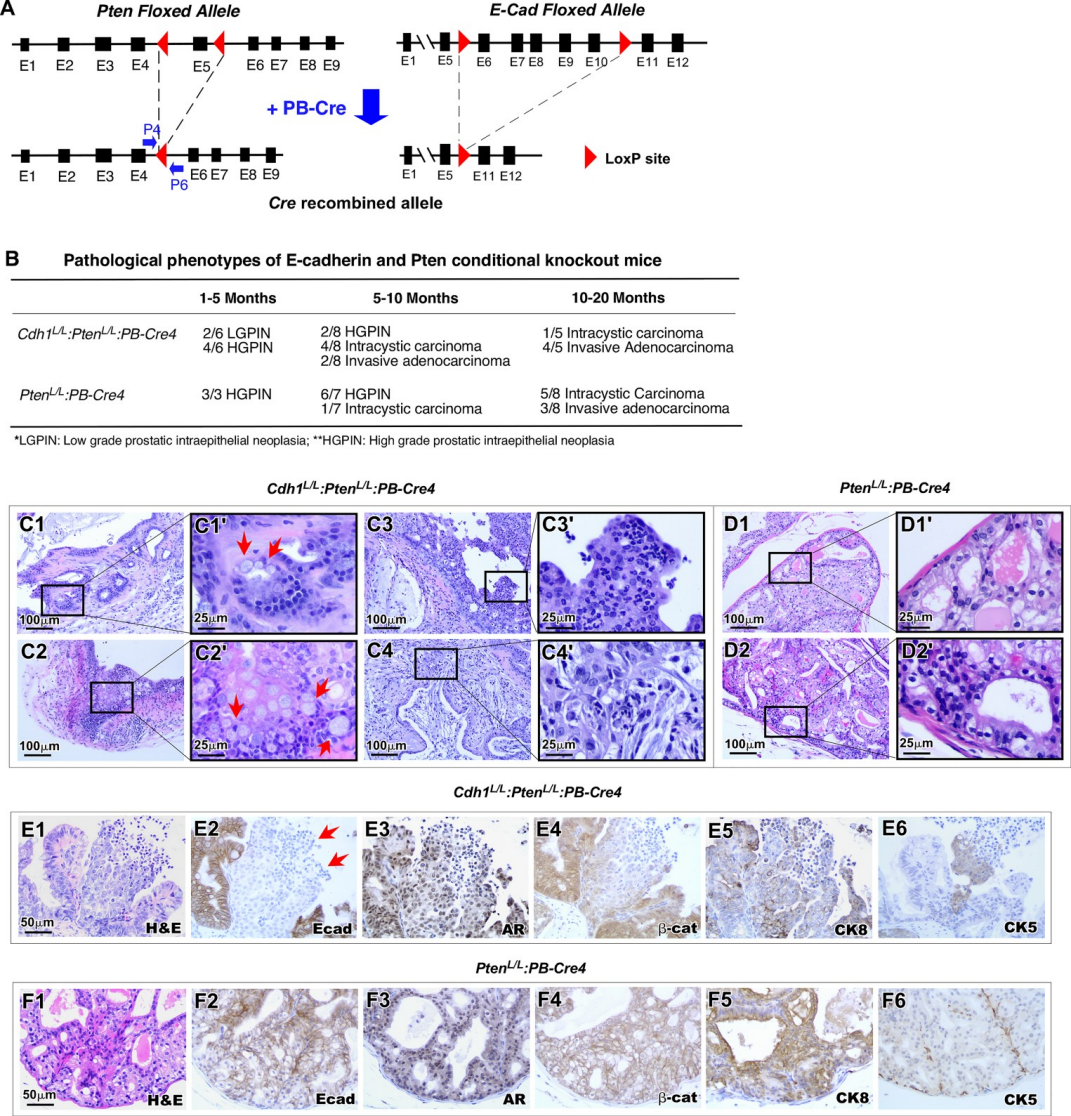
E-cadherin-Pten bigenic knockout results in increased invasion, inflammation & goblet cell metaplasia in the prostate. **A.** Gene construct of the targeted *Pten* floxed allele in addition to the previously described *Cdh1* targeted allele undergoing recombination in the presence of *PB-Cre4*. **B.** Table summarizing the pathological phenotypes observed in *Cdh1*^*L/L*^:*Pten*^*L/L*^:*PB-Cre4* vs *Pten*^*L/L*^:*PB-Cre4* control mice by age. **C1-C4’** Representative images of key pathological features observed in 5–10 month old *Cdh1*^*L/L*^:*Pten*^*L/L*^:*PB-Cre4* mice. Pathological features include invasive adenocarcinoma with goblet cell metaplasia indicated by arrows (C1’). Red arrows indicate areas with goblet cell metaplasia (C1’and C2’). **D1-D2’** Representative images of typical PIN features observed in 5–10 month old *Pten*^*L/L*^:*PB-Cre4* control mice. **E1-F6.** Representative images of H&E and IHC staining using the indicated antibodies from adjacent prostate tissue sections from *Cdh1*^*L/L*^:*Pten*^*L/L*^:*PB-Cre4* and *Pten*^*L/L*^:*PB-Cre4* mice. Red arrows indicate E-cadherin negative cells (E2). Scale bars, located in the bottom left corner of images, are sized as indicated.

Loss or reduction of E-cadherin has been shown to occur frequently in many advanced and invasive human tumors, and directly contributes to tumor progression [12,13]. Aberrant PI3K signaling alterations have been observed in advanced and metastatic prostate cancers [34,35]. Recent studies further suggest that loss of E-cadherin and PI3K activation co-exist in human tumors and play a collaborative role in tumor progression [36]. To directly assess the role of E-cadherin deletion in tumor progression, we generated *Cdh1*^*L/L*^:*Pten*^*L/L*^:*PB-Cre4* compound mice (Fig 6A), in which deletion of the tumor suppressor *Pten* and *Cdh1* simultaneously occur in mouse prostatic epithelium. We observed accelerated prostate cancer development and progression as well as early onset of inflammation in compound mice in comparison to *Pten*^*L/L*^:*PB-Cre4* mice (Fig 6B and 6C1-4’ versus 6D1-2’). In addition to histological lesions reflecting earlier prostatic tumor invasion (Fig 6C1-1’), various pathologies were also shown in tumor samples of the compound mice, including aberrant differentiation (Fig 6C2-2’), transepithelial leukocyte migration (Fig 6C3-3’), and pools of extracellular mucin spilling into the stromal compartment (Fig 6C4-4’). IHC analysis showed E-cadherin negative tumor cells (red arrows, Fig 6E2) surrounded by E-cadherin expressing cells within tumor lesions of the compound mice. Expression of the AR and CK8 appeared in both E-cadherin positive and negative tumor cells (Fig 6E3 and 6E5). However, CK5 staining presented predominately in E-cadherin negative tumor cells (Fig 6E6). Cytoplasmic staining of β-catenin was also observed in E-cadherin negative cells in contrast to clear membrane staining observed in E-cadherin positive cells (Fig 6E4 and 6F4). In *Pten*^*L/L*^:*PB-Cre4* mice, most prostatic tumor cells were stained positively for E-cadherin, AR, and CK8 (Fig 6F2, 6F3 and 6F5, respectively) while CK5 positive cells were mainly observed lining the basement membrane areas (Fig 6F6). Taken together, the development of an early onset and aggressive tumor phenotype in *Cdh1*^*L/L*^:*Pten*^*L/L*^:*PB-Cre4* compound mice in comparison with *Pten*^*L/L*^:*PB-Cre4* mice demonstrates a promotional role of E-cadherin deletion in prostate tumor formation and progression.

### Development of invasive adenocarcinoma with mucinous features in the prostate of *Cdh1*^*L/L*^:*Pten*^*L/L*^:*PB-Cre4* mice

**Fig 7:**
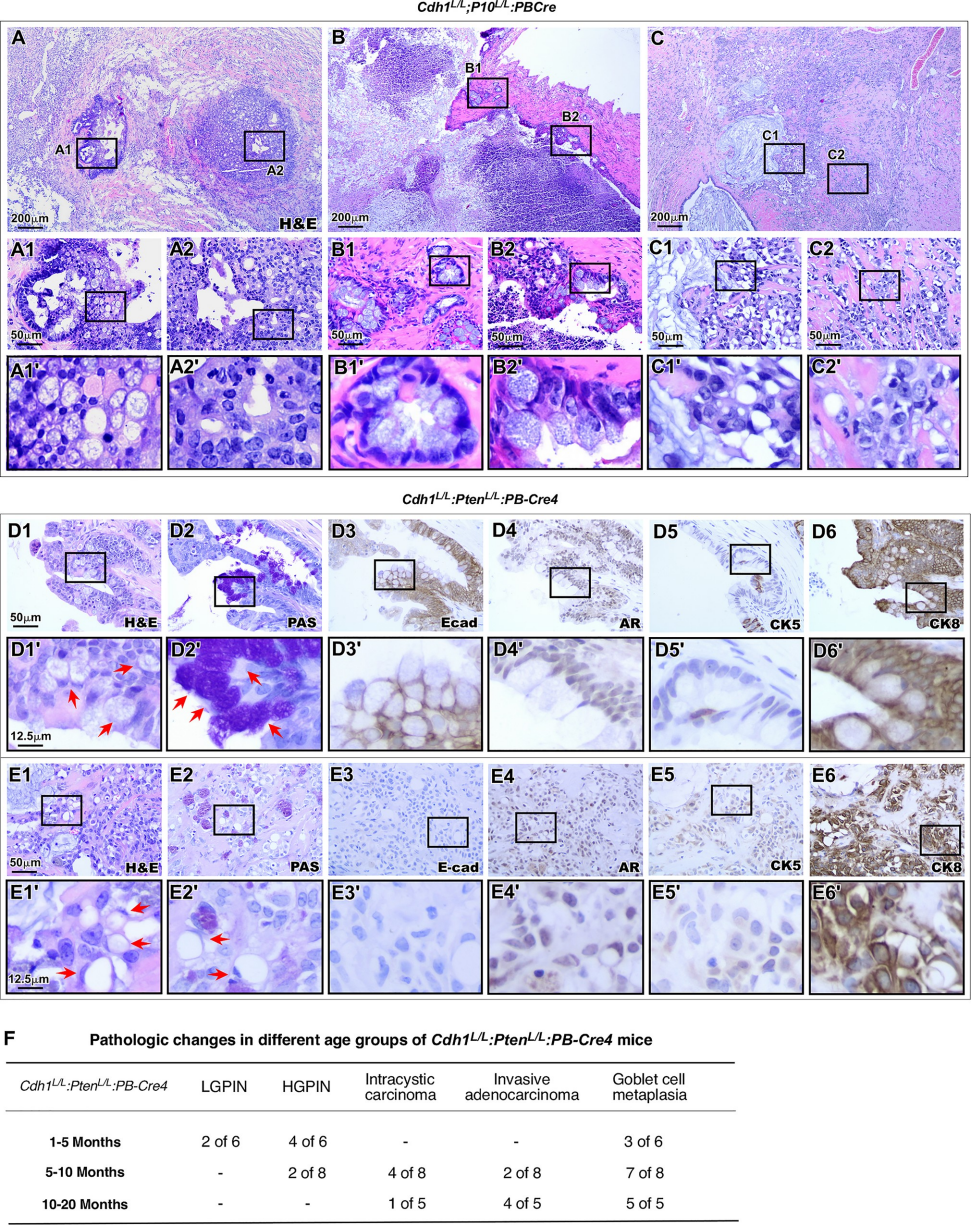
Goblet cell metaplasia is associated with increased invasion and tumor aggression in E-cadherin-Pten bigenic knockout mouse prostates. **A-C2’.** H&E images of prostate tissue sections from *Cdh1*^*L/L*^:*Pten* ^*L/L*^:*PB-Cre4* mice between 15 & 20 months of age. **D1-D6’.** Representative images of goblet cell metaplasia using various stains as indicated. Arrows denote PAS positive goblet cells (D1’ and D2’). **E1-E6’.** Representative images characterizing the expression pattern observed in adenocarcinoma cells with goblet cell differentiation. Arrows denote signet ring cells observed amongst invasive E-cadherin negative epithelial cells (E1’ and E2’). **F.** Table summarizing pathological observations in *Cdh1*^*L/L*^:*Pten* ^*L/L*^:*PB-Cre4* mice according to age. Scale bars, located in the bottom left corner of images, are sized as indicated.

As age progressed, *Cdh1*^*L/L*^:*Pten*^*L/L*^:*PB-Cre4* compound mice further developed more aggressive tumor phenotypes with a variety of pathological changes. Importantly, we observed a distinct population of cells resembling goblet cells admixed with different prostatic tumor lesions. As shown in Fig 7A to 7C, goblet cell metaplasia presented in lesions with adenocarcinoma *in situ*, invasive adenocarcinoma, and invasive mucinous carcinoma. While prostatic adenocarcinoma lesions presented both with and without goblet cell metaplasia (Fig 7A1 and 7A2 respectively), increased numbers of goblet cells appeared in early invasive tumor lesions (Fig 7B1-1’) and in more extensively invasive lesions with pools of extracellular mucin (Fig 7C1-2’), suggesting an association between the presence of goblet cells and tumor invasiveness and aggressiveness. High magnification visualization of these tumor lesions showed typical goblet cell morphology (arrows, Fig 7D1’). These goblet cells feature large mucin filled vacuoles displacing the nucleus to the cell periphery [37] and were observed in 12 of 13 (~92%) *Cdh1*^*L/L*^:*Pten*^*L/L*^:*PB-Cre4* mice over 5 months of age (Fig 7F). To evaluate the status of neutral mucinous components within the goblet cells, we performed Periodic acid-Schiff (PAS) staining. Clear positive staining of PAS appeared in goblet cells (red arrows, Fig 7D2’). Interestingly, staining for E-cadherin still appeared in most prostatic goblet cells (Fig 7D3-3’). Weak AR staining was shown in most goblet cells and the majority of goblet cells were stained positive for CK8 as well. Interestingly, goblet cells and the majority of the surrounding epithelium stained negative for CK5 indicating the loss of the basal layer of the epithelium in these regions (Fig 7D4-6’). In more severe cases, invasive carcinomas with prominent goblet and signet ring cells were observed as shown in Fig 7E1-6’. These invasive lesions contained extracellular mucin along with intracellular mucin vacuoles (Fig 7E1-E2’). In additional, PAS negative signet ring cells (SRCs) were observed within these lesions (arrows, Fig 7E1 and 7E2). Furthermore, these highly aggressive lesions stained completely negative for E-cadherin while maintaining positive AR and CK8 staining (Fig 7E3, 7E4 and 7E6). Only sparse CK5 staining was observed in mucinous carcinoma lesions (Fig 7E5–7E5’). Taken together, we demonstrate that simultaneous deletion of E-cadherin and Pten in mouse prostate epithelium promotes tumor cell transdifferentiation and invasiveness, and produces more aggressive tumor phenotypes with goblet and signet ring cell infiltration.

## Discussion

In this study, we evaluated the biological role of E-cadherin in the mouse prostate using newly generated genetically engineered mouse models. As detailed above, *Cdh1*^*L/L*^:*PB-Cre4* mice developed pathological lesions that are very similar to PIN as early as 6 weeks of age but failed to develop invasive prostatic tumor lesions. Intriguingly, in addition to the above PIN lesions, an increase in epithelial denudation and apoptotic cells also presented in prostatic epithelium of *Cdh1*^*L/L*^:*PB-Cre4* mice. These data suggest that loss of E-cadherin in prostatic epithelial cells not only leads to oncogenic transformation and growth but also disrupts epithelial organization and induces cell apoptosis and tissue damage. With these two distinct fates, E-cadherin deletion cells appear insufficient to progress and form prostate tumors as we observed in *Cdh1*^*L/L*^:*PB-Cre4* mice. These new lines of scientific evidence implicate a dynamic role of E-cadherin in prostatic epithelium during the course of prostate cell oncogenic transformation and growth. Although the molecular mechanisms for PIN failing to progress to prostate tumors in this mouse model need to be further investigated, increased cell apoptosis and tissue damage in E-cadherin deletion cells suggest that additional abnormalities and mutations are required for E-cadherin negative atypical cells to survive, progress and form prostate tumors.

Since E-cadherin complexes with the actin cytoskeleton via cytoplasmic catenins to maintain the functional characteristics of epithelia [38], it has been shown that loss of E-cadherin resulted in increased cytoplasmic and nuclear levels of β-catenin in tumor cells [14]. In addition, lost or reduced expression of E-cadherin has been shown to disrupt this complex in advanced and poorly differentiated prostate cancers [25]. In this study, we performed a series of experiments to examine whether the cadherin-bound pool of β-catenin can be released and participate in the canonical Wnt-signaling pathway and act as a co-activator of the AR [29,30]. We showed that knockdown of E-cadherin expression elevated free cytoplasmic and nuclear β-catenin levels, and increased androgen-induced transcription activity in prostate cancer cells. Intriguingly, knockdown of either β-catenin or AR expression in the above experiments significantly attenuated androgen-induced transcriptional activity. Using both cell proliferation and colony formation assays, we further demonstrated a promotional role of E-cadherin knockdown in enhancing cell growth and colony formation in the above prostate cancer cells. These data implicate that loss of E-cadherin increases the level of cytoplasmic and nuclear β-catenin and enhances the activity of the AR-mediated transcription and cell growth. In accordance with the above results, we also observed elevated expression of cytoplasmic and nuclear β-catenin in E-cadherin deleted atypical cells within PIN lesions in *Cdh1*^*L/L*^:*PB-Cre4* mice. Moreover, an increase in both Cyclin D1 and Myc expression, the downstream targets of β-catenin, also appeared in the above E-cadherin deleted cells. Since mutations in β-catenin, APC, and other components of the destruction complex appear very rarely in prostate cancer cells [17–19], our data from both *in vitro* and *in vivo* experimental approaches provide scientific evidence to elucidate a novel molecular mechanism underlying E-cadherin in regulating β-catenin cellular localization, and activity in promoting oncogenic transformation and cell growth in prostate cancer cells.

In this study, in addition to PIN development, increased cell apoptosis was also observed in prostatic epithelium of *Cdh1*^*L/L*^:*PB-Cre4* mice. The anoikis of prostatic luminal epithelial cells appeared both in prostate tissues and organoids derived from prostatic epithelial cells of *Cdh1*^*L/L*^:*PB-Cre4* mice. An increase in prostatic intermediate cells also presented in the above tissue and organoid samples, implicating prostatic luminal cell replenishment by increasing basal to luminal epithelial cell differentiation. The above pathological changes appeared much more obviously in younger *Cdh1*^*L/L*^:*PB-Cre4* mice at less than three months of age. Observation of co-existing PIN lesions and cell anoikis in the prostate of the E-cadherin deletion mice is novel and interesting. The above two distinct cellular events have actually been reported in two separate studies previously [10] [33]. Temporal deletion of E-cadherin using Nkx3.1 driven Cre-ER showed apoptotic cell death mainly via anoikis and increased vertical divisions from prostatic basal to luminal cells, but no PIN formation [10]. These results are consistent with our observations and implicate the critical role of E-cadherin in maintaining epithelial integrity in the prostate. In a similar study, the development of PIN and prostate cancer was observed in the mouse line with conditional deletion of E-cadherin using Probasin-Cre [33], but interestingly, no anoikis in prostate tissues was observed. Although the reasons causing the above discrepancy between our study and the previous report are unclear, the pagetoid distribution of E-cadherin deleted cells within PIN lesions in our current mouse model provide scientific evidence to demonstrate the nature of these cells being physically restrained within the epithelium in order to survive. The presence of E-cadherin positive cells within PIN lesions of the current *Cdh1*^*L/L*^:*PB-Cre4* mice further implicate luminal cell replenishment and mosaic recombination occurring during the course of PIN and cellular apoptosis events. In this study, using *R26*^*mTmG/+*^:*Cdh1*^*L/L*^:*PB-Cre4* mouse strain, we also confirmed mosaic deletion of *Cdh1* in prostatic epithelial cells. These multiple lines of evidence demonstrate an essential role of E-cadherin in maintaining prostate epithelial integrity for epithelial differentiation and growth.

Loss of E-cadherin expression has been observed in advanced and metastatic prostate cancer [25]. To assess the biological role of E-cadherin loss in prostate cancer progression, we generated the *Cdh1*^*L/L*^:*Pten*^*L/L*^:*PB-Cre4* compound mouse model, in which both E-cadherin and Pten deletion co-occur in mouse prostate epithelium. Given the biological significance of PI3K signaling pathway alterations in prostate cancer, this new bigenic deletion mouse model can also address a collaborative role of Pten and E-cadherin loss in prostate cancer initiation and progression. An accelerated PIN and prostate tumor development was observed in *Cdh1*^*L/L*^: *Pten*^*L/L*^:*PB-Cre4* compound mice in comparison to *Pten*^*L/L*^:*PB-Cre4* only mice. Pathological analysis of *Cdh1*^*L/L*^:*Pten*^*L/L*^:*PB-Cre4* compound mice showed a variety of pathologic changes, reflecting more aggressive tumor phenotypes. In addition to invasive prostatic adenocarcinoma lesions observed in *Pten*^*L/L*^:*PB-Cre4* mice, *Cdh1*^*L/L*^:*Pten*^*L/L*^:*PB-Cre4* compound mice also showed poorly differentiated prostatic invasive mucinous carcinomas. The presence of infiltrating myeloid-derived suppressor cells (MDSCs) has been reported in prostatic tumors of *Pten*^*L/L*^:*PB-Cre4* mice [39]. Interestingly, we observed an apparent increase in the number of MDSCs in tumor lesions of *Cdh1*^*L/L*^:*Pten*^*L/L*^:*PB-Cre4* compound mice compared to *Pten*^*L/L*^:*PB-Cre4* mice (S2A1-2 Fig and S2B1-2 Fig). An increase in cell numbers with positive staining for MDSC markers, Cd11b and Gr-1, was also revealed in prostatic tumor lesions of *Cdh1*^*L/L*^:*Pten*^*L/L*^:*PB-Cre4* compound mice compared to *Pten*^*L/L*^:*PB-Cre4* mice (S2A3-4 Fig and S2B3-4 Fig). In support of these observations, increased expression of iNOS and Arg1, two genes commonly expressed by MDSCs, were also observed in prostatic tumor tissues of the *Cdh1*^*L/L*^:*Pten*^*L/L*^:*PB-Cre4* compound mice in comparison with either *Pten*^*L/L*^:*PB-Cre4 or Cdh1*^*L/L*^:*PB-Cre4* mice (S2C Fig). Moreover, elevated activation of PI3K/AKT pathways also appeared in tumor samples of the compound mice in comparison with those of *Pten*^*L/L*^:*PB-Cre4 or Cdh1*^*L/L*^:*PB-Cre4* mice. Furthermore, increased expression of anti-apoptotic regulator, Bcl-2, was also observed in prostate tissue samples of *Cdh1*^*L/L*^:*Pten*^*L/L*^:*PB-Cre4* mice compared to those of *Cdh1*^*L/L*^:*PB-Cre4 or Pten*^*L/L*^:*PB-Cre4* mice (S2H Fig). These data provide more scientific insight into the molecular basis for the promotional role of E-cadherin loss in Pten-deficient prostatic tumor progression. The above mouse models will provide the useful tools for more in-depth mechanistic investigation in the future.

Most notably from this study, we identified goblet cell metaplasia within tumor lesions of *Cdh1*^*L/L*^:*Pten*^*L/L*^:*PB-Cre4* compound mice. Goblet cell related tumor lesions are relatively rare and have not been frequently observed in prostate mouse models. The goblet cells feature large mucin filled vacuoles displacing the nucleus to the cell periphery [37]. The presence of prostatic goblet cell metaplasia in this mouse model was accompanied by inflammatory lesions, cystic prostate gland formation, transepithelial leukocyte migration, and extracellular pools of mucin. Interestingly, most goblet cells identified in prostatic tumor lesions appeared to retain E-cadherin expression, suggesting that goblet cells do not originate from E-cadherin deletion cells but rather arise through other mechanisms during the course of tumor progression. It will be significant and interesting to further assess the potential effect of inflammatory responses in goblet cell metaplasia development using the current mouse models.

Taken together, results presented in this study implicate a critical role of E-cadherin in prostate morphogenesis and tumorigenesis. Our data show that loss of E-cadherin expression not only induces oncogenic transformation and PIN development through elevating cytoplasmic and nuclear β-catenin expression and activity but also disrupts normal epithelial structure and cell-cell contacts resulting in apoptosis and inflammatory responses. In combination with Pten deletion in the mouse prostate, loss of E-cadherin results in an early onset and more aggressive prostate tumor development. Importantly, more aggressive pathological changes with prostatic goblet cell metaplasia are featured in the prostate tumor lesions in the compound mice. Further investigation using these new mouse models should provide fresh insight into the effect of E-cadherin loss in prostate development, morphogenesis, and tumorigenesis.

## Material and methods

### Ethics statement

All experimental procedure and care of animals in this study were carried out according to the Institutional Animal Care and Use Committee (IACUC) at Beckman Research Institute at City of Hope, and approved by the IACUC. Euthanasia was performed by CO2 inhalation followed by cervical dislocation.

### Mouse mating and genotyping

The mice used in this study were all from a C57BL/6 background. The *Cdh1* floxed mice (*Cdh1*^*L/L*^) were kindly provided by Dr. Rolf Kemler [9]. The *PB-Cre4* mice were obtained from the NCI mouse repository (strain #: 01XF5*)*. *Rosa26*^*mTmG/+*^ (*R26*^*mTmG/+*^) reporter mice were kindly provided by Dr. Liqun Luo [40]. The *Pten* floxed mice (*Pten*^*L/L*^) were kindly provided by Dr. Hong Wu [41]. Experimental mice were generated by intercrossing *Cdh1*^*L/L*^ females with *Cdh1*^*L/L*^:*PB-Cre4* males. Similar mating procedures were used to generate *R26*^*mTmG/+*^:*Cdh1*^*L/L*^:*PB-Cre4* and *R26*^*mTmG/+*^:*Cdh1*^*L/L*^ mice. The *Cdh1* and *Pten* bigenic knockout mice were generated by mating *Cdh1*^*L/L*^:*Pten*^*L/L*^ females with *Cdh1*^*L/L*^:*Pten*^*L/L*^:*PB-Cre4* males. Genotyping was performed as previously described [42–44]. DNA samples collected from different prostatic lobes and tissues of *Cdh1*^*L/L*^:*PB-Cre4* and *Cdh1*^*L/L*^ mice, including testis, liver, kidney, bladder and tail were used. For the detection of the recombined *Cdh1* allele, the forward primer (5’- GAATTCTGAACATCATTATCAGTATTTA- 3’) and reverse primer (5’-TGACACATGCCTTTACTTTAGT- 3’) were used with the following conditions: 94°C for 2 min, 94°C for 30’, 52°C for 45’, 72°C for 45’. Detection of the *Cdh1* targeted allele used forward primer, 5’-CTTATACCGCTCGAGAGCCGG-3’ and reverse primer, 5’-GTGTCCCTCCAAATCCGATA-3’. Detection of the *Pten* allele used the forward primer, 5’-TCCCAGAGTTCATACCAGGA-3’ with the reverse primer, 5’-AATCTGTGCATGAAGGGAAC-3’. For the *R26*^*mTmG/+*^ alleles the forward primer, 5’-TCAATGGGCGGGGGTCGTT-3’ was used with reverse primers, 5’-CTCTGCTGCCTCCTGGCTTCT-3’ and 5’-CGAGGCGGATCACAAGCAATA-3’. Lastly, detection of the PB-Cre4 allele used forward primer, 5’-GATCCTGGCAATTTCGGCTAT-3’ and reverse primer, 5’-GCAGGAAGCTACTCTGCACCTTG-3’. Genomic PCR conditions for targeted alleles were amplified at 95°C for 3 min, then 95°C for 45 sec, 58°C for 40 sec, and 72°C for 60 sec for 35 cycles, then 72°C for 5 min.

### Pathological analyses

Mouse tissue samples were fixed in 10% neutral-buffered formalin and processed in paraffin or processed to OCT following cryo-protection in 30% sucrose in 1X PBS pH 7.3 at 4°C overnight as previously described [45,46]. Following embedding in paraffin or OCT, tissue blocks were cut to 4 μm serial sections and used for hematoxylin-eosin (H&E) staining for further pathological analyses [46]. Pathological analyses were performed in accordance with the guidelines recommended by The Mouse Models of Human Cancers Consortium Prostate Pathology Committee in 2013 [23].

### Immunohistochemistry (IHC), Immunofluorescence (IF), PAS and PAS/D, mTmG, and TUNEL assays

IHC was performed following rehydration of tissue sections through a decreasing ethanol gradient. Antigen retrieval was performed using a microwave to boil the slides in 0.01M citrate buffer (pH 6.0) followed by 15 min in 0.3% H_2_O_2_ in methanol. Tissue sections then underwent blocking for 1 hr at room temperature in 5% goat serum in 1X PBS (pH 7.3) and incubated with primary antibodies in 1% goat serum in PBS overnight at 4°C. Tissues sections were washed with PBS and then incubated with biotinylated secondary antibodies for 1 hr at room temperature in 1% goat serum in PBS. Then, slides were incubated with Streptavidin ligated to horse radish peroxidase (Strep-HRP) (SA-5004, Vector Laboratories, 1:500 dilution) for 30 min and developed with 3,3'-diaminobenzidine (DAB) kit (SK-4100, Vector Laboratories). Tissue sections were counterstained in 5% Harris Hematoxylin and dehydrated through an increasing ethanol gradient. Coverslips were mounted using Permount Medium (SP15-500, Fisher Scientific) as described previously [47].

Immunofluorescence staining was performed using the similar procedures as described above for IHC, but excluding the use of H_2_O_2_ in methanol. Slides were developed with fluorescent-conjugated secondary antibodies and then mounted with coverslips using Vectashield Mounting Medium with DAPI (H-1200, Vector Laboratories). The mTmG assay was performed as previously described [48]. Briefly, sections from OCT embedded tissues were washed with PBS, pH 7.3, and then developed with Vectashield Mounting Medium with DAPI (H-1200, Vector Laboratories). For the TUNEL assay, Click-iT Plus TUNEL Assay Kit (C10617, Invitrogen) was used according to the manufacturer protocol (MAN0010877). Antibodies used for both IHC and IF are listed (S1 Table).

PAS staining was performed as described earlier [49]. Briefly, PAS/D sections were treated with 1% diastase (AHDIA50, American MasterTech) in H_2_O for 30 min and 0.5% periodic acid (19840–0050, Acros) for 5 min at room temperature. After rinsing with H_2_O, Schiff’s reagent (26312-1A, Electron Microscopy Sciences) was added for 30 min at room temperature. Slides were rinsed in lukewarm water and counterstained with hematoxylin. Slides were dehydrated through an increasing ethanol gradient and coverslips were mounted using Permount Medium (SP15-500, Fisher Scientific).

### Microscope image acquisition

Images of immunohistochemistry and H&E staining were taken using an Axio Lab A1 microscope using 5x, 10x, 20x and 40x Zeiss A-Plan objectives. A Canon EOS 1000D camera and axiovision software (Carl Zeiss, Oberkochen, Germany) were used to capture images. Fluorescence imaging was performed using a Nikon ECLIPSE E800 Epi-Fluorescence Microscope using 20x and 40x Nikon Plan Fluor objectives with a QImaging RETIGA EXi camera and using QCapture software (QImaging). Three-dimensional z-stack imaging was performed using a Zeiss Inverted LSM700 Microscope with Zen 2012 imaging software for acquisition and analysis.

### Organoid culture

Primary prostate tissue was collected from *R26*^*mTmG/+*^:*Cdh1*^*L/L*^:*PB-Cre4* and *R26*^*mTmG/+*^:*Cdh1*^*L/L*^ littermates at 6 weeks of age. Mouse prostate tissues were minced and then dissociated to single cells by digestion using collagenase/hyaluronidase (C/H) & TrypLE (12605–028, Gibco). Digested cells were resuspended in Dulbecco's Modified Eagle's Medium (DMEM)/Ham’s F-12 50/50 Mix (DMEM/F12) plus 10% FBS, penicillin-streptomycin, Y-27632 dihydrochloride and dihydrotestosterone (DHT) and approximately 30,000 cells per well were plated with Matrigel (BD Biosciences, #356231) into 24-well plates. Cells were cultured in DMEM/F12 with B7, N-acetylcysteine, EGF, Noggin, R-spondin1, A83-01, Y-27632 and DHT, as previously described [50], for 3 days before changing to fresh medium for the last two days. Following the 5-day culture, organoids were collected, washed with PBS and fixed in 10% neutral-buffered formalin before being embedded in Histogel (HS-4000-012, ThermoFisher) and undergoing paraffin embedding for histological analysis. For full mount analysis, organoids, following fixation, were suspended in 30% sucrose.

### Cell cultures, lentivirus and adenovirus production, and Transient transfections

The human embryonic kidney cell line, HEK293, was maintained in Dulbecco's modified Eagle's medium (DMEM) supplemented with 5% fetal calf serum (FCS) (HyClone, Denver, CO). Human prostate cancer cell lines, LNCaP and LAPC4, were maintained as described previously [32]. Transient transfections were carried out using a Lipofectamine 2000 kit (Invitrogen, Carlsbad, CA). Approximately 1.5x10^4^ cells were seeded into a 48-well plate 16 hrs before transfection. Approximately 300 ng of total plasmid DNA and 0.5 μl of Lipofectamine-2000 per well were used in the transfection as previously described [51]. To generate shRNA lentiviruses, pLenti-shRNA vectors, pCMV-dR8.91, and pMD2.G-VSVG plasmids were co-transfected into HEK293T cells at a ratio of 3:2:1 using a Lipofectamine kit as described previously [52,53]. The media were replaced at 6 hrs post-transfection and then collected 36 to 40 hrs later. The viral supernatant was centrifuged briefly to remove cellular debris and stored at -80°C. Lentivirus infection was carried out in the presence of 6 mg/ml Polybrene [54,55].

### Preparation of the cytosolic fraction and nuclear extracts

LNCaP cells were cultured for making nuclear extracts and cytosolic fractions as described in the previous report [56]. Nuclear extracts were prepared essentially according to the method of Dignam *et al* with minor modifications [57]. Briefly, the cells were washed with PBS and mechanically disrupted by scraping into homogenization buffer A (10mM Hepes pH 7.9, 10mM KCl, 1.5mM MgCl2, 0.5mM DTT, and 0.5mM PMSF) and incubated on ice for 10 min. Cells were further disrupted by 10 strokes of a homogenizer and centrifuged at 15,000 rpm for 20 min. The pellet was resuspended in buffer containing 20mM Hepes pH 7.9, 420mM NaCl, 1.5mM MgCl2, 0.2mM EDTA, 0.5mM DTT, 0.5mM PMSF, and 25% glycerol, and then homogenized with 10 strokes. The lysate was incubated on ice for 30min and centrifuged for 10 min at 15,000 rpm. The supernatant was saved and analyzed as the nuclear fraction. To prepare the cytosolic fraction, LNCaP cells treated with LY294002 were lysed in digitonin lysis buffer (1% digitonin, 150mM NaCl, 50mM Tris-HCl pH 7.5, 10mM MgCl2). The lysates were centrifuged at 13,000 rpm for 10 min, and supernatants were saved as cytosolic components. The pellets representing cytoskeletal and nuclear components were lysed in RIPA buffer.

### Plasmid construction

The pPSA7kb-luc plasmid was obtained from Dr. Jan Trapman [58,59]. A CMV-driven β-galactosidase (β-gal) reporter was generated by cloning the lacZ gene into the pcDNA3 vector [32,60]. Double-stranded oligonucleotides corresponding to the *CDH1* sequences 5’-GGCACAGATGGTGTGATTACAGA-3’, 5’-GGCTGGACCGAGAGAGTTTCCCA-3’and 5’-GGCGAGTGCCCAACTGGACCAA-‘3 were synthesized and cloned into the pLenti-shRNA vectors, for making the E-cadherin shRNA1 shRNA2 and shRNA3 constructs respectively [52,53].

### Transfection, luciferase and β-gal assays

Transient transfections were carried out using LipofectAMINE 2000 (Invitrogen). For androgen induction experiments, cells were grown in T-medium or RPMI with charcoal-stripped fetal calf serum (HyClone) for 16 to 24 hr in the presence or absence of 1 nM DHT. After an 18–24 hr incubation, cells were harvested, and the luciferase and β-gal activities were measured. The RLU from individual transfections was normalized using β-gal activity in the same samples. Individual transfection experiments were done in triplicate and the results are reported as mean RLU/β-gal (±SD).

### Cell proliferation and colony formation

Approximately 2000 cells per well were plated and cultured as described previously [32]. Proliferation assays were carried out using the MTS (3-(4,5-dimethylthiazol-2-yl)-5-(3-carboxymethoxyphenyl)-2-(4-sulfophenyl)-2H-tetrazolium) tetrazolium kit (Promega, Madison, WI). Cell numbers were determined by absorbance at 490 nm as suggested by the manufacturer. For colony formation assay, LNCaP cells were plated in 6-well plates, approximately 500 cells/well for 24 hr, and then cultured for 10–12 days. The size and number of colonies were determined by staining with crystal violet (Sigma, St. Louis, MO).

### SDS-PAGE and immunoblotting

Protein fractions for immunoblotting were boiled in SDS-sample buffer and then resolved on a 10% SDS-PAGE. The proteins were transferred onto a nitrocellulose membrane and probed with appropriate antibodies, including anti-E-cadherin (mouse, 1:2500, #610181, BD Transduction Laboratories), anti-β-Tubulin (mouse, 1:2000, #581P1702F, NeoMarkers). Proteins were detected using the ECL kit (Amersham, Arlington Heights, IL). The nuclear fractions were analyzed by SDS-PAGE.

### RNA Isolation and Reverse Transcription-Quantitative PCR (RT-qPCR)

RNA samples were isolated from fresh mouse prostate tissues using RNeasy Mini Kit (Qiagen, Cat #74104). Reverse transcription was performed using SuperScript IV First-Strand Synthesis System (ThermoFisher, Cat # 18091050) and RT-qPCR assays were carried out using PowerSYBR Green PCR Master Mix (4367659, Applied Biosystems) with specific primers (S2 Table) on the 7500 Real-Time PCR system (Thermo Fisher Scientific).

### RNA sequencing analysis

RNA sequencing analysis was performed using publically available raw sequencing data from GEO accession number GSE115204 [33]. Using DNAnexus, raw fastq files were mapped using STAR Mapping (1.3.3). The data was then mapped to the (GRCm38) mm10 reference genome using BowTie2 FASTQ Read Mapper (1.4.1). Genome resource archive was built using the Tuxedo Protocol Resource Builder (1.1.4). Gene expression results were then prepared using Cuffdiff (1.0.6). Differential gene expression was evaluated using edgeR via web app (https://luyangcoh.shinyapps.io/RNAseq_Diff_analysis/) kindly provided by Lu Yang in Beckman Research Institute at City of Hope. The database for annotation, visualization, and integrated discovery software (DAVID v6.8) was used along with Gene Set Enrichment Analysis (GSEA 3.0) in order to identify processes that were up or down regulated in *Cdh1* floxed mice compared to wild-type mice based on differential gene expression. All presented processes were identified to be statistically significant with a P-value less than 0.05.

### Statistical analyses

Statistical analyses were performed using 2-tailed Student’s t test. The date was presented as the means ± S.D and cell numbers were made comparisons between groups, using a Student’s *t* test. *p* < 0.05 were considered significant.

## Supporting information

S1 Fig Generating *R26^mTmG/+^:Cdh1^L/L^:PB-Cre4* mice. A scheme demonstrates co-occurring recombination of the *Cdh1* allele and the *R26*^*mTmG*^ reporter allele driven by *PB-Cre4* expression.
(PDF)

S2 Fig Molecular basis for promotional role of E-cadherin knockout in Pten deficient mouse prostate tissue. **A1-B4.** Representative images of H&E and IHC staining of myeloid derived suppressor cells (MDSCs) in prostate tissue sections from *Cdh1*^*L/L*^:*P10*^*L/L*^:*PB-Cre4* and *P10*^*L/L*^:*PB-Cre4* mice. **C.** RT-qPCR results comparing expression levels of the indicated genes between *Cdh1*^*L/L*^:*PB-Cre4*, *P10*^*L/L*^:*PB-Cre4* and *Cdh1*^*L/L*^:*P10*^*L/L*^:*PB-Cre4* mice. **D1-G5.** Representative images of H&E staining and IHC staining of PI3k-Akt pathway genes in the indicated genotypes. **H.** RT-qPCR results comparing the expression levels of apoptosis related gene, Bcl-2, between *Cdh1*^*L/L*^:*PB-Cre4*, *P10*^*L/L*^:*PB-Cre4* and *Cdh1*^*L/L*^:*P10*^*L/L*^:*PB-Cre4* mice. Scale bars, located in the bottom left corner of images, are sized as indicated.
(PDF)

S1 Table Antibodies used for IHC and IF staining (see the “Material and Methods” section also). (PDF)

S2 Table Primers used for RT-qPCR for S2 Fig (see the “Material and Methods” section also). (PDF)
