# Supplementary material for: The comprehensive role of E-cadherin in maintaining prostatic epithelial integrity during oncogenic transformation and tumor progression
Source: PLoS Genet. 2019 Oct 28;15(10):e1008451. doi: 10.1371/journal.pgen.1008451 (PMC6816545; doi:10.1371/journal.pgen.1008451)
Supplement: S1 Fig — A scheme demonstrates co-occurring recombination of the Cdh1 allele and the R26mTmG reporter allele driven by PB-Cre4 expression. (PDF) [file pgen.1008451.s001.pdf]

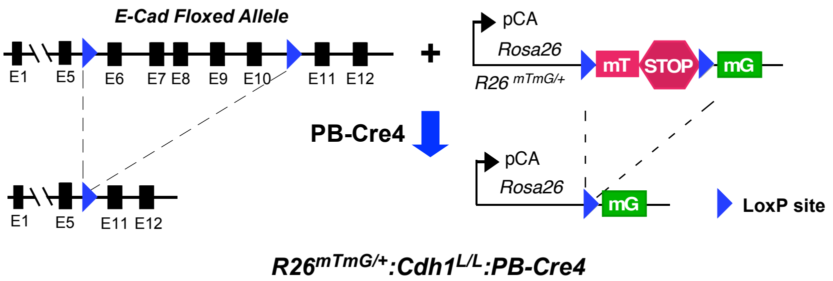

S1 Fig. GENERATING *R26$^{mTmG/+}$*:*CDH1$^{L/L}$*:*PB-CRE4* MICE. A scheme demonstrating co-occurring recombination of the *Cdh1* allele and the *R26^{mTmG}* reporter allele driven by *PB-Cre4* expression.
