# Supplementary material for: The comprehensive role of E-cadherin in maintaining prostatic epithelial integrity during oncogenic transformation and tumor progression
Source: PLoS Genet. 2019 Oct 28;15(10):e1008451. doi: 10.1371/journal.pgen.1008451 (PMC6816545; doi:10.1371/journal.pgen.1008451)
Supplement: S2 Fig — A1-B4. Representative images of H&E and IHC staining of myeloid derived suppressor cells (MDSCs) in prostate tissue sections from Cdh1L/L:P10L/L:PB-Cre4 and P10L/L:PB-Cre4 mice. C. RT-qPCR results comparing expression levels of the indicated genes between Cdh1L/L:PB-Cre4, P10L/L:PB-Cre4 and Cdh1L/L:P10L/L:PB-Cre4 mice. D1-G5. Representative images of H&E staining and IHC staining of PI3k-Akt pathway genes in the indicated genotypes. H. RT-qPCR results comparing the expression levels of apoptosis related gene, Bcl-2, between Cdh1L/L:PB-Cre4, P10L/L:PB-Cre4 and Cdh1L/L:P10L/L:PB-Cre4 mice. Scale bars, located in the bottom left corner of images, are sized as indicated. (PDF) [file pgen.1008451.s002.pdf]

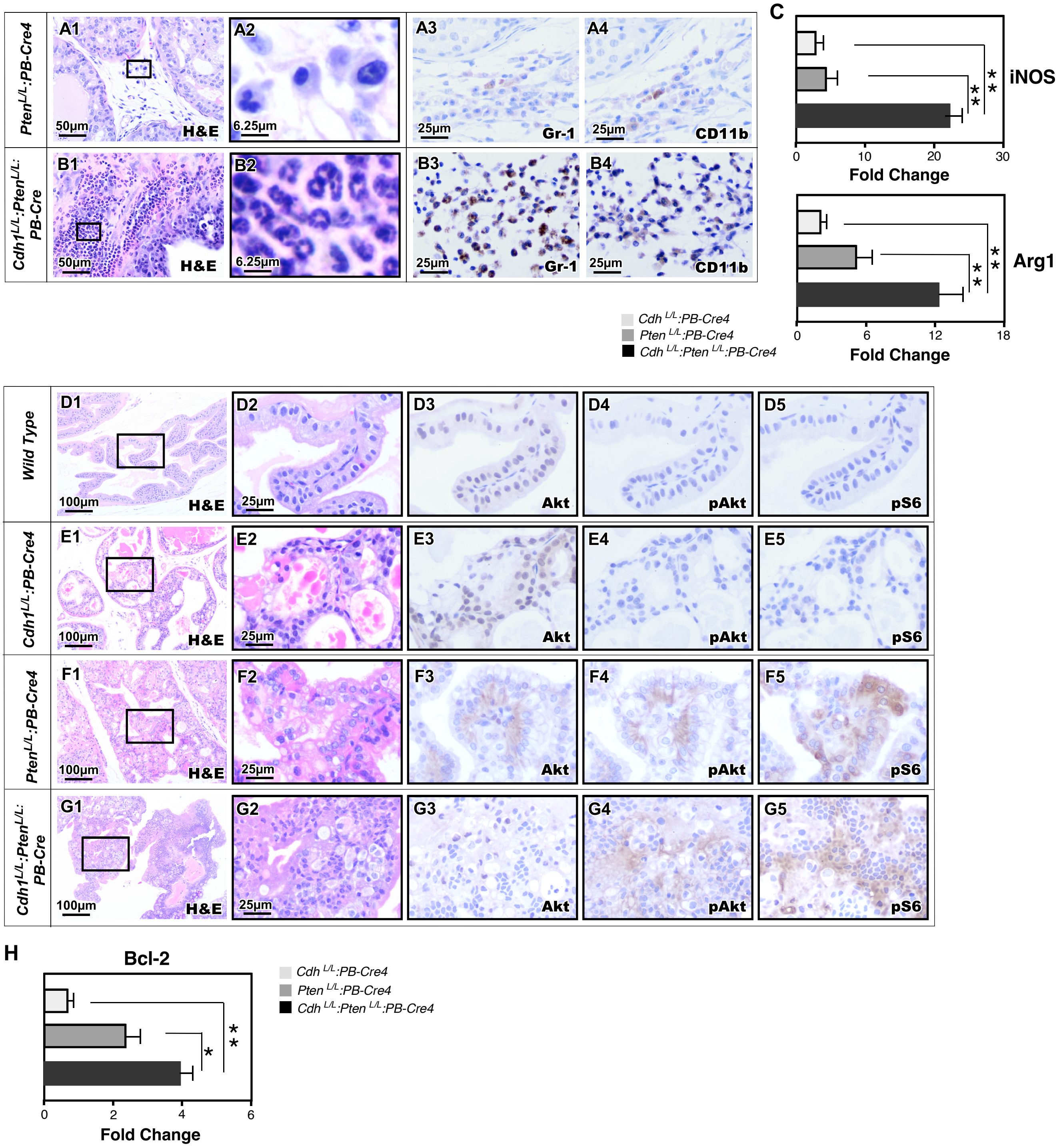

S2 Fig MOLECULAR BASIS FOR THE PROMOTIONAL ROLE OF E-CADHERIN KNOCKOUT IN PTEN DEFICIENT MOUSE PROSTATE TISSUE. A1-B4. Representative images of H&E and IHC staining of myeloid derived suppressor cells (MDSCs) in prostate tissue sections from *Cdh1*$^{L/L}$*:P10*$^{L/L}$*:PB-Cre4* and *P10*$^{L/L}$*:PB-Cre4* mice. **C.** RT-qPCR results comparing expression levels of the indicated genes between *Cdh1*$^{L/L}$*:PB-Cre4, P10*$^{L/L}$*:PB-Cre4* and *Cdh1*$^{L/L}$*:P10*$^{L/L}$*:PB-Cre4* mice. **D1-G5.** Representative images of H&E staining and IHC staining of PI3k-Akt pathway genes in the indicated genotypes. **H.** RT-qPCR results comparing the expression levels of apoptosis related gene, Bcl-2, between *Cdh1*$^{L/L}$*:PB-Cre4, P10*$^{L/L}$*:PB-Cre4* and *Cdh1*$^{L/L}$*:P10*$^{L/L}$*:PB-Cre4* mice. Scale bars, located in the bottom left corner of images, are sized as indicated.
