# Supplementary material for: The comprehensive role of E-cadherin in maintaining prostatic epithelial integrity during oncogenic transformation and tumor progression
Source: PLoS Genet. 2019 Oct 28;15(10):e1008451. doi: 10.1371/journal.pgen.1008451 (PMC6816545; doi:10.1371/journal.pgen.1008451)
Supplement: S1 Table — (PDF) [file pgen.1008451.s003.pdf]

S1 Table   ANTIBODIES USED FOR IHC & IF STAINING

Antibody	Vendors and Cat#	Species	Working Dilution
E-cadherin	Cell Signaling Technology, #3195	Rabbit IgG	1:200
E-cadherin	BD Transduction Laboratories, #610182	Mouse IgG	1:200
GFP	Cell Signaling Technology, #2955	Mouse IgG	1:250
CK5	Covance, PRB-160P	Rabbit IgG	1:2000
CK8	Biolegend, #904801	Mouse IgG	1:2000
PCNA	Santa Cruz Biotechnology, sc-56	Mouse IgG	1:200
AR	Invitrogen, #PA5-16750	Rabbit IgG	1:500
β-catenin	BD Transduction Laboratories, 610154	Mouse IgG	1:500
β-catenin	Santa Cruz Biotechnology, sc-7199	Rabbit IgG	1:500
CyclinD1	Abcam, #ab16663	Rabbit IgG	1:200
Ki67	Cell Signaling Technology, #9129	Rabbit IgG	1:500
Myc	Abcam, #ab32072	Rabbit IgG	1:100
Cd11b	Abcam, #ab8878	Rat IgG	1:100
Gr-1	R&D Systems, MAB1037	Rat IgG	1:100
Akt	Cell Signaling Technology, #9272	Rabbit IgG	1:100
pAKT	Cell Signaling Technology, #3787	Rabbit IgG	1:50
pS6	Cell Signaling Technology, #2211	Rabbit IgG	1:50
Biotinylated anti-rabbit	Vector Laboratories, #BA-1000	Goat IgG	1:750
Biotinylated anti-mouse	Vector Laboratories, #BA-9200	Goat IgG	1:750
Goat anti-rabbit 488	Invitrogen #A11034	Goat IgG	1:500
Goat anti-mouse 488	Invitrogen #A11001	Goat IgG	1:500
Goat anti-rabbit 594	Invitrogen #A11012	Goat IgG	1:500
Goat anti-mouse 594	Invitrogen #A11005	Goat IgG	1:500
