# Supplementary material for: The comprehensive role of E-cadherin in maintaining prostatic epithelial integrity during oncogenic transformation and tumor progression
Source: PLoS Genet. 2019 Oct 28;15(10):e1008451. doi: 10.1371/journal.pgen.1008451 (PMC6816545; doi:10.1371/journal.pgen.1008451)
Supplement: S2 Table — (PDF) [file pgen.1008451.s004.pdf]

S2 Table PRIMERS USED FOR RT-QPCR

RT-qPCR	Gene	Primer	Sequence
	iNOS	Forward	CAGCTGGCCAATGAGGTACT
		Reverse	GTGCCAGAAGCTGGAACTCT
	Arg1	Forward	AAGAAAAGGCCGATTCACCT
		Reverse	CACCTCCTCTGCTGTCTTCC
	Bcl2	Forward	GTCCCGCCTCTTCACCTTTCAG
		Reverse	GATTCTGGTGTTTCCCCGTTGG
